# Effects of topical Vancomycin Dressing on Methicillin-Resistant *Staphylococcus Aureus* (MRSA) positive diabetic foot ulcers

**DOI:** 10.12669/pjms.35.4.368

**Published:** 2019

**Authors:** Anas Bin Saif, Sohail Jabbar, Muhammad Saeed Akhtar, Ahmed Mushtaq, Mansoor Tariq

**Affiliations:** 1 Dr. Anas Bin Saif, MBBS, FCPS Consultant General Surgeon, Department of Surgery, Combined Military Hospital, Kohat, Pakistan; 2 Dr. Sohail Jabbar, MBBS, FCPS Consultant General Surgeon, Department of Surgery, Combined Military Hospital, Kohat, Pakistan; 3 Dr. Muhammad Saeed Akhtar, MBBS, FCPS, FACS Consultant General and Laparoscopic Surgeon, HoD Surgical Department, Department of Surgery, Combined Military Hospital, Kohat, Pakistan; 4 Dr. Ahmed Mushtaq, MBBS, FCPS, OJT Consultant General and Orthopedic Surgeon, Department of Surgery, Combined Military Hospital, Kohat, Pakistan; 5 Dr. Mansoor Tariq, MBBS, FCPS Consultant General Surgeon, Department of Surgery, Combined Military Hospital, Kohat, Pakistan

**Keywords:** Diabetic foot ulcer, MRSA, Vancomycin

## Abstract

**Objective:**

To compare the effects of simple saline dressings versus topical vancomycin dressings on Methicillin-resistant *Staphylococcus Aureus* positive chronic diabetic foot ulcers.

**Methods:**

It was quasi experimental study conducted in Combined Military Hospital Kohat and PNS-Shifa Hospital Karachi from 01 January 2017 to 31 December 2017. A total of 23 patients were included based on non-probability convenient sampling who had diabetes and presented with foot ulcers for more than two weeks showing positive growth of Methicillin-Resistant *Staphylococcus Aureus*. The patients were treated with simple saline soaked dressings and debridement at first for three weeks followed by three weeks of topical vancomycin dressings with debridement. Thus patients served as their own controls

**Results:**

The average change in surface area with saline dressing was +1.73 ±1.53cm^2^ per week whereas with vancomycin soaked dressing it was --0.06±1.60 cm^2^ per week (p <0.05). The average exudate also decreased from 1.78±1.23 to 0.99±0.72 (p<0.05) and same trend was observed in percentage of slough covering the ulcer from 45% ± 22.3% to 24.3% ±12.90% (p<0.05) with vancomycin dressing. Moreover, fifteen patients had negative culture for MRSA within 2 weeks.

**Conclusion:**

Vancomycin impregnated dressing in MRSA positive Diabetic foot may help achieve early healing as compared to simple conventional dressings with no systemic toxicity.

## INTRODUCTION

Globally it is estimated that 382 million people suffer from diabetes with a prevalence of 8.3%. In Pakistan prevalence of this devastating disease is 6.8%.^1^ Diabetic foot is defined as a foot affected by ulceration that is associated with neuropathy and/or peripheral arterial disease of the lower limb in a patient with diabetes. Diabetic foot infections range in severity from superficial paronychia to deep infection involving bone. Types of infection include cellulitis, myositis, abscesses, necrotizing fasciitis, septic arthritis, tendinitis, and osteomyelitis. It is estimated that about 5% of all patients with diabetes present with a history of foot ulceration, while the lifetime risk of diabetic patients developing this complication is 15%.^2^,^3^ Foot ulceration and infection are the leading risk factors for amputation.^4^ This puts a great emphasis on early and accurate management of diabetic foot ulcer. Treatment of diabetic foot involves a multimodal approach including debridement of the wound, management of any infection, revascularization procedures when indicated, glycemic control and off-loading of the ulcer. Other methods have also been suggested to be beneficial as add-on therapies, such as hyperbaric oxygen therapy, use of advanced wound care products, and negative-pressure wound therapy (NPWT).

Debridement is one of the gold standards in wound healing management, significantly contributing to the healing process of the wound, including the diabetic ulcer.^5^ Available data on the use of dressings in diabetic wounds is limited still there is evidence of their role in prevention of infection and enhancement of wound healing.^6^,^7^

Most ulcers have multi-microbial growth, however, Staphylococcus Aureus is most common. Methicillin Resistant *Staphylococcus Aureus* (MRSA) also possess a serious threat to Diabetic foot ulcers (DFU) as 30-50% ulcers have MRSA positive strain.^8^ Studies done in our country shows that burden of disease is very high in our setup due to poor glycemic control and lack of awareness. A large number of patients present with high grade diabetic wound in our clinics.^9^,^10^

Therefore, management of diabetic foot ulcers remains a major therapeutic challenge for our surgeons especially MRSA positive DFUs, which implies an urgent need to review strategies and treatments in order to achieve the goals and reduce the burden of care in an efficient and cost-effective way. So we designed this study to see effects of topical vancomycin dressing on MRSA positive diabetic foot ulcers.

## METHODS

After ethical approval from ethical committee of hospital this quasi experimental study was planned from 01 Jan 2017 to 31 Dec 2017. All the patients suffering from diabetic foot ulcer for more than 02 weeks and having MRSA positive strain were included. Patients suffering from sepsis, osteomyelitis, allergic to vancomycin and those who did not gave written informed consent were excluded from the study. The data was collected by non-probability convenient sampling technique. The patients were treated at first with saline dressings and debridement twice weekly for first three weeks then the same wound was treated with vancomycin impregnated dressing for next three weeks.^11^,^12^ Thus patients served as their own controls. Vancomycin dressing was made by mixing 500mg of injection in 5 ml Saline and spraying it on dry gauze placed in wound. The patients were not given any parenteral or oral antibiotics.

The variables measured apart from demographic data were change in surface area, amount of exudate and amount of slough covering the wound. Area of wound was calculated by linear method, multiplying greatest length to greatest width in centimeters after sharp debridement. Area was calculated at the end of week subtracting it from previous calculated area to get the change in surface area and mean change in area was calculated for simple dressing at end of third week by adding the change in area per week and dividing it by 3. Similar technique was applied for vancomycin dressing. The exudate was assessed by scoring system where 0= none, 1= scanty, 2=some or minimal, 3=moderate and 4=frank pus.^11^ SPSS Inc. version 17 was used for data analysis. Mean and Standard deviation was calculated for each variable. Paired t-test was applied to calculate the p-value. Value less than 0.05 was taken as significant keeping confidence interval of 95%.

## RESULTS

A total number of 23 patients were included in the study. There were 15 (65%) males and 8 (35%) female subjects. Average age of males was 59 ± 5.78 years and females was 55 ± 4.66 years (cumulative age was 57 ± 7.72 years). Average time for which patients had diabetes was 10 ± 4.5 years. At the time of presentation 22 (95%) patients had peripheral neuropathy, 14 (61%) had peripheral vascular disease, 10 (43%) had other co-morbids and 5 (22%) had some form of malnutrition.

**Fig. 1:**
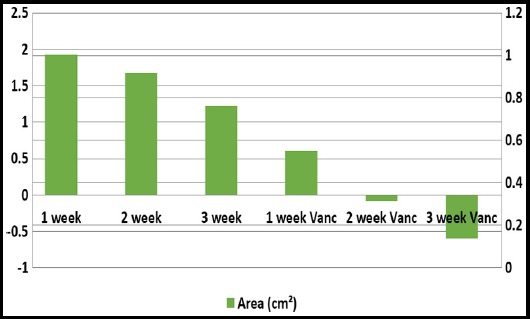
Bar graph showing average change in surface area per week

**Fig. 2:**
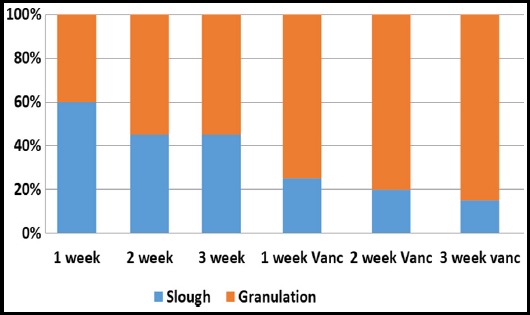
Bar graph showing percentage of slough and granulation per week.

The average change in size per week is shown in Fig.1. There was a net increase in surface area of 1.93 cm^2^ in first week followed by 1.68 and 1.22cm^2^. However, after third week, when vancomycin was added to dressings, the surface area of wounds started to decrease from average 0.6 cm^2^ to -0.08 cm^2^ and 0.6cm^2^ in last week. The net change in amount of slough covering the surface of wound is shown in Fig.2. There was net decrease of 15 % slough using saline dressing in first three weeks, whereas, with vancomycin dressings the percentage granulation increased by 30% in next three weeks. Similarly, the amount of exudate was objectively assessed and the exudate at end 3^rd^ week was 1.78 ± 1.23 with use of saline dressing and when vancomycin was added in the following 3 weeks the average exudate was decreased to 0.99 ± 0.72. The comparison in terms of change in surface area, amount of exudate and percentage of slough covering the wound between simple dressings and vancomycin dressings is shown in Table-I. There is a significant statistical difference between the two groups in terms of these variables depicted by p-value <0.05. Moreover 15 (65%) patients had negative cultures after 2 weeks of using vancomycin dressing. There was no systemic toxicity or allergy reported in all cases.

**Fig. 3:**
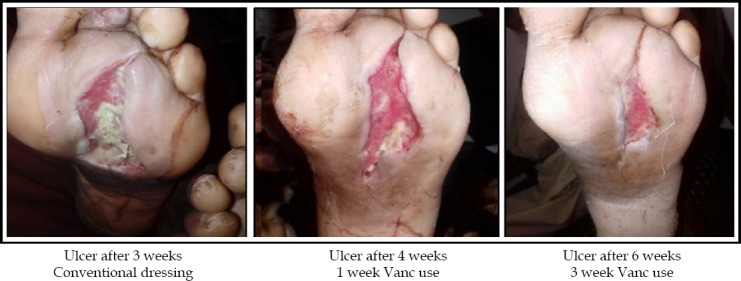
Showing change in surface area in 63 years old male patient.

**Table I:** Showing the change in surface area, amount of exudate and percentage slough comparison between two groups.

S. No	Variable	Simple Dressing	Vancomycin dressing	p-Value (paired t-test)
1	Change in surface area	+1.73 ±1.53cm^2^	-0.06±1.60 cm^2^	< 0.05
2	Amount of exudate	1.78±1.23	0.99±0.72	<0.05
3	Percentage of slough	45% (±22.3%)	24.3% (±12.90%)	<0.05

## DISCUSSION

Infections especially of foot in diabetic patients can have serious outcomes if not managed properly. Another emerging serious concern is development of resistance to antibiotics especially in chronic wounds as DFU.^13^ Our study showed a cumulative age of 57 ± 7.72 years and the duration of diabetes was 10 ± 4.5 years at which the patients develop DFU. The age and duration at which patients of diabetes develop DFU is less as compared to international studies.^14^ Globally foot ulcers are common in diabetics with age over 60 years and having diabetes for 12 or more years.^15^ Relatively young age and early development of DFUs in our population can be due to lack of awareness and lack of access to better diagnostic facilities.

The most common cause of DFU in our study was neuropathy followed by peripheral arterial disease which is consistent with internationally published studies.^16^ Our study showed that male diabetic patients develop DFU more commonly (65% males). The study conducted by Amjad SS and colleagues showed that male to female ratio for DFUs was 2:1 which is consistent with our study.^17^ The male preponderance can be due to mal-foot ware and frequent out door traveling. In our study the MRSA positive strain was present in 20% of all DFUs with *Staphylococcus Aureus* as most common organism isolated in 55% of cases. In Pakistan, MRSA positive strains have been found in 20-40% of DFUs^18^ whereas internationally 30-50% of patients with DFU have MRSA strain.^5^ The most common single organism isolated from DFUs is *Staphylococcus Aureus* which was also observed in our case.^19^

This is the first ever study conducted in Pakistan to encompass the treatment of DFU in MRSA positive strains. Various antibiotics have been studied for topical use in DFUs as metronidazole, neomycin, polymyxin B but vancomycin has not been studied extensively.^20^,^21^ Our findings depicted that there was significant decrease in exudate and average change in surface area by application of vancomycin impregnated dressings as compared to simple dressing (p-value <0.01). There was also a rapid decline in the percentage of slough in wound with simultaneous increase in granulation tissue which almost doubled from 15% to 30% (p-value<0.01) when vancomycin dressing was used, pointing to the fact that topical vancomycin may help in early wound healing. Moreover, the bacterial culture was also negative for MRSA after two weeks of application in 65% cases. There was also no hypersensitivity reported in our study with vancomycin. A randomized controlled trial of 426 patients showed that triple antibiotic topical application significantly reduces infection rate in minor wounds.^21^ Albaugh KW et al. studied the effects of vancomycin on chronic wounds and found out that topical applications reduce the bacterial count and may promote healing which was also found in our study.^11^ Whereas Simons et al showed no role of topical antibiotics in severe wound infections in head and neck surgeries.^22^ There are many advantages of using topical antibiotics as high and sustainable concentration of antibiotic available at site of infection, limited total amount of antibiotic needed, limited possibility for systemic toxicity, may prevent development of resistance as systemic drug is not given and it can be applied as OPD case. However, there are some disadvantages as well like minimal penetration to surrounding tissue limits its ability to be used in more severe infection, only few preparations are available, there is possible risk of allergic reaction and there is risk of possible alteration of normal skin flora.^21^ Since the patients were not given any parental or enteral antibiotics so it is worthwhile to apply topical antibiotic application while avoiding systemic use.

There were a few limitations in our study. Sample size is small and data was compared of only one part of country so its result cannot be generalized to whole population. Moreover, the patients were serving as their own controls and were treated with saline dressings first so there is a possibility of procedural bias. Moreover, the cost effect relationship of use of vancomycin was not studied. The author recommends a multi-center randomized controlled trial to evaluate the efficacy of topical vancomycin use is MRSA positive strains.

## CONCLUSION

The use topical preparation of vancomycin in MRSA positive strains in chronic diabetic foot wounds help in significantly reducing the average surface area, amount of exudate and percentage of slough covering the wound as compared to conventional saline soaked dressings. It also simultaneously causes significant decrease in positive MRSA cultures and may promote early healing. The systemic toxicity is also reduced when vancomycin is applied topically.
